# Supplementary material for: Quantitative Risk Assessment for the Introduction of Carbapenem-Resistant Enterobacteriaceae (CPE) into Dutch Livestock Farms
Source: Antibiotics (Basel). 2022 Feb 21;11(2):281. doi: 10.3390/antibiotics11020281 (PMC8868399; doi:10.3390/antibiotics11020281)
Supplement: Supplementary file 1 [file antibiotics-11-00281-s001.zip › antibiotics-1575401-supplementary.pdf]

antibiotics

Article

Quantitative Risk Assessment for the Introduction of Carbapenem-Resistant Enterobacteriaceae (CPE) into Dutch Livestock Farms

Natcha Dankittipong [1,*], Egil A. J. Fischer [1], Manon Swanenburg [2], Jaap A. Wagenaar [3], Arjan J. Stegeman [1] and Clazien J. de Vos [2]

[1] Department Population Health Sciences, Farm Animal Health, Utrecht University, Martinus G. de Bruingebouw, Yalelaan 7, 3584 CL Utrecht, The Netherlands; e.a.j.fischer@uu.nl (E.A.J.F.); j.a.stegeman@uu.nl (J.A.S.)
[2] Wageningen Bioveterinary Research, Wageningen University & Research, Houtribweg 39, 8221 RA Lelystad, The Netherlands; manon.swanenburg@wur.nl (M.S.); clazien.devos@wur.nl (C.J.d.V.)
[3] Department Biomolecular Health Science, Infectious Diseases & Immunology, Utrecht University, Androclusgebouw, Yalelaan 1, 3584 CL Utrecht, The Netherlands; j.wagenaar@uu.nl
* **Correspondence:** n.dankittipong@uu.nl

Supplementary Materials

Supplementary File S1: Literature review

Publications related to Carbapenemase-producing Enterobacteriaceae were searched through Pubmed with the following search strings:

carbapenem OR carbapenems OR carbapenemase OR carbapenemase-producing OR carbapenemase producing OR carbapenem resistance OR carbapenem-resistant OR carbapenem resistant OR carbapenemase-positive OR VIM OR KPC OR OXA OR NDM

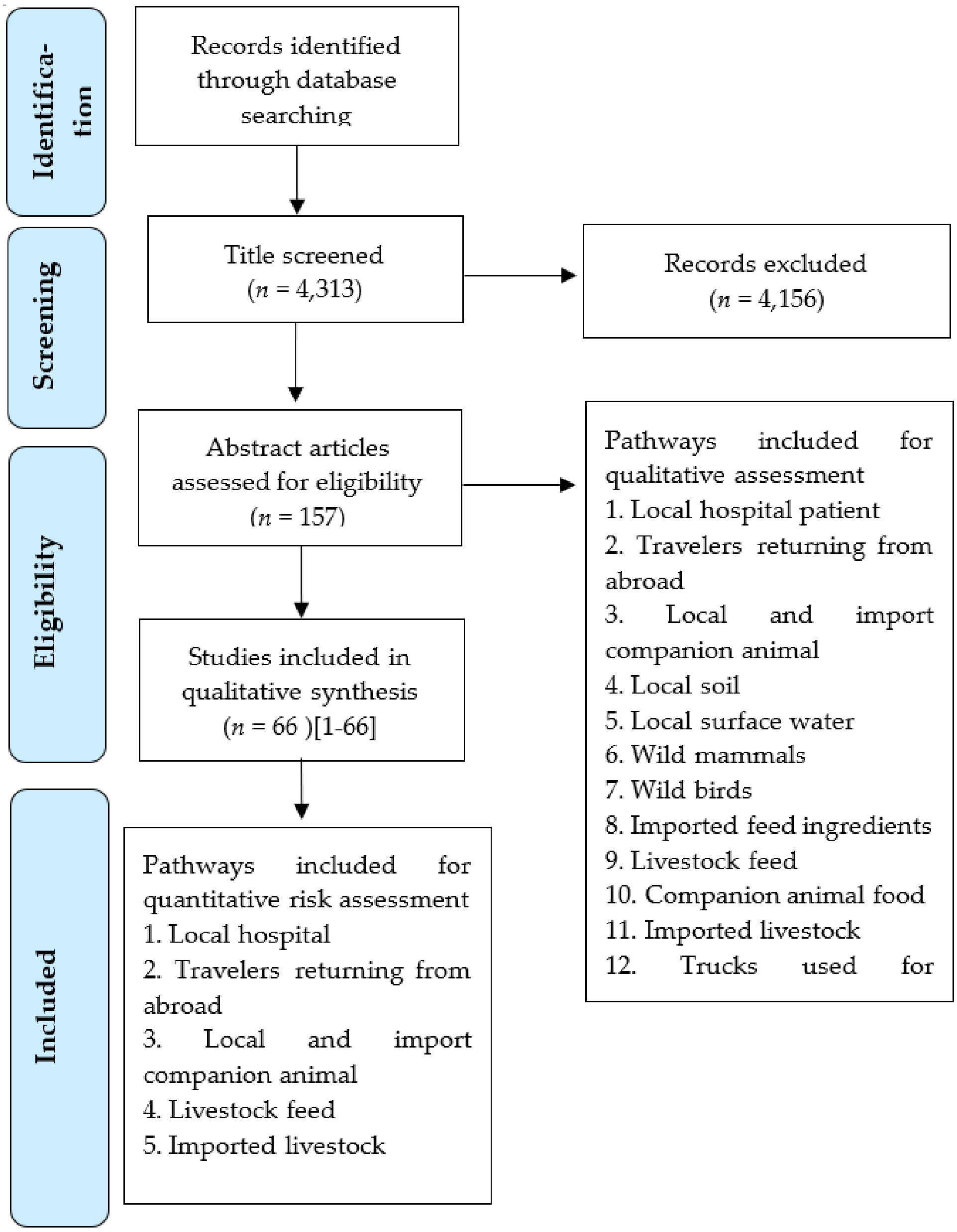

Figure S1. PRISMA chart indicates the literature review of potential CPE sources.

Supplementary File S2: Report expert elicitation in projects "Risk assessment CPE" and "BEWARE".

Expert elicitation in 3 rounds:

1. Open questions about "reservoirs" of CPE for exposure of Dutch livestock and companion animals
2. Conjoint analyses going more detailed into reservoirs and different regions of origin
3. Workshop to work out pathways in more detail

1. Open questions about "reservoirs" of CPE for exposure of Dutch livestock and companion animals

Aim: Make an inventory of possible reservoirs of CPE (a reservoir is a

Method: Expert names were provided by Dik Mevius and other MRA experts from WBVR. Experts were approached by email. Two questions were asked:

In the first question a list of possible CPE reservoirs, described in literature, was given. In this question the experts were asked if the list was complete. If they thought the list was not complete, they were asked to mention additional possible

reservoirs.
The second question was "Which 3 reservoirs do you consider the most important for introduction of CPE in (Dutch*) livestock/companion animals?". Experts could give an explanation (but were not obliged to do so).
Results: Ten experts sent their answers.

Question 1:
The original list of possible reservoirs in the question:
- Sewage water
- Waste water from waste water treatment plants
- Waste (water) from hospitals,
- Waste (water) from industry
- Animal feed
- Travellers
- Manure
- Imported livestock
- Imported pets
- Imported animal products
- Imported fish, seafood, shellfish

Additional reservoirs mentioned by the experts were (in random order):
- Wild birds, fresh surface water, imported products (vegetables, fruits, spices),
- humans in The Netherlands (not only travellers), animals (not only imported ones)
- pets, animal feed, humans,
- human population, not only imported animal sources, wildlife
-immigrants' workers and foreign guest workers, wildlife and other environmental sources, environmental bacteria
- you forgot the humans!!
- Dutch residents
- tentatively, treated domestic pigs, human carriers of CPE who work with livestock and poultry

Question 2:
Counting which reservoirs were mentioned as most important by the experts resulted in the following table.

Most important routes by the experts	How many times in top 3?	
**Animals**		
imported livestock	3	
imported animals	3	
imported animals if not screened	1	
imported animal products	2	
imported pets	1	
pets	1	
		11
**Humans**		
travellers	3	

humans	1	
humans, including travellers	1	
human carriers	2	
travellers including guest workers	1	
human population (including inhabitants and travellers/visitors)	1	
humans (working in livestock production)	1	
		10
**Waste water**		
waste water from hospitals	3	
waste water	1	
sewage water or sewage related such as aerosols	1	
waste water from WWTPs	1	
		6
**wildlife**		1
**animal feed**		1
**raw feed derived from risk countries** (the expert means pet food)		1
Total		**30**

Discussion:

It seems that not all experts had understood that, in question 2, they had to think of reservoirs for animals to become infected. Perhaps it was not completely clear to all experts (although it was explained) that we asked for the introduction risk for exposure of animals (and not for The Netherlands or humans).

In the list of reservoirs that we sent to the experts, we only mentioned humans in the form of travellers. The experts added many different human categories as a possible reservoir.

Conclusion "Open questions about CPE reservoirs":

The experts estimated imported livestock and other animals as most important reservoir, followed by all kind of humans and waste water.

The most important reservoir added by the experts to the original list was humans. These were people having been hospitalized in The Netherlands, and immigrant workers.

2. Conjoint analyses going more detailed into reservoirs and different regions of origin

Aim: to get more insight in the most important reservoirs and regions of origin for possible exposure of Dutch animals with CPE. As the results of part 1 indicated importance of humans, and imported animals, we tried to differentiate more within these categories.

Method:

A questionnaire was sent by the participating experts. This consisted of 3 parts.

In the first part the experts were asked to look at 20 comparisons of 3 combinations of a possible reservoir for CPE's and a region in the world from which this reservoir originates. For each comparison, experts had to **choose the combination** of reservoir and region which, in their opinion, **leads to the highest probability of exposure of Dutch animals** (livestock, pets) to CPE.

In the second part, the experts were shown two times 8 comparisons. In 2A they had to compare different animals as reservoir, and in 2B different types of humans. Each comparisons showed 3 types of animals (2A) or humans (2B). Experts were asked to choose the reservoir (animal or "type of human") which they considered the **most important** for introduction of CPE, leading to exposure of Dutch livestock/companion animals, ánd the animal or human that is **least important**.
In the third part experts had to divide 100 points over reservoirs (3A) and regions (3B), so that relative importance of each reservoir or region is shown (more points when more important).
The results of part 1 and 2 were analysed with XLStat in Excel (part 1 in choice based conjoint analysis; part 2 in Maxxdiff analysis). The results of part 3 were put in Excel and average number of added points per reservoir and region were calculated. Reservoirs and regions were ranked, based on averages of points given, but also based on the average of rank numbers per expert.

Results:
Eight experts participated in this step of the expert elicitation.

Part 1: The results showed that regions were considered as more important than reservoirs (68% vs 32%). The experts considered Asia as the region with the highest risk, followed by Africa and southern Europe. The reservoir leading to the highest risk was "waste water from hospitals", according to the experts, followed by "humans travelling from abroad to The Netherlands" and "water from waste water treatment plants".

Part 2: The comparison of "different types of humans" as a reservoir resulted in the following ranking:
1. People from abroad immigrating to the NL
2. People from abroad coming to work for a period in the NL
3. People from the NL returning from travel abroad
4. Dutch residents that have recently been or are in hospital or other healthcare institutions
5. People from abroad visiting the NL for holiday/business

The comparison of different imported animals as a reservoir resulted in the following ranking:
1. Veal calves
2. Dogs
3. Pigs
4. Poultry
5. Horses
6. Cats

Part 3: This part was a "check" for consistency of the experts with the answers given in part 1 and 2.
Here, 100 points were divided over reservoirs and regions.
We looked at the results in 2 ways: First we calculated the average of the points given by the eight experts per reservoir and per region. That resulted in the following ranking order of reservoirs and regions.

Reservoir	Average	Total
Humans travelling from abroad to the Netherlands	21.25	170

Waste water from hospitals	16.88	135
Water from waste water treatment plants	14.25	114
Imported animal products	12.88	103
Imported livestock	12.63	101
Travelling pets	11.25	90
Humans hospitalized	10.88	87
Total	100.00	

Region	Average	Total
Asia	27.6	221
Africa	19.1	153
Southern Europe	15.5	124
Eastern Europe	11.4	91
Southern America	10.3	82
Western Europe	5.8	46
Northern America	5.6	45
Oceania	3.0	24
Northern Europe	1.75	14
Total	100.0	

Another way of analysing this part was making a ranking per expert, based on the points given (reservoir with most point was ranked 1, and so on), and then we calculated the average rank number. This resulted in:

Reservoir	Rank
Waste water from hospitals	1
Humans travelling from abroad to the Netherlands	2
Imported animal products	3
Travelling pets	4
Imported livestock	5
Waste water from waste water treatment plants	6
Humans hospitalized	7

Region	Rank
Asia	1
Africa	2
Southern Europe	3
Southern America	4
Eastern Europe	5
Northern America	6
Western Europe	7
Oceania	8
Northern Europe	9

We noticed some differences between the results with the different way of analysing. The most important difference is the rank of "waste water from waste water treatment plants". This was ranked 3 based on average points and rank 6, based on rank number. This was caused by one expert who appointed many points to this reservoir.
For the regions the top 3 and the least important regions did not differ between analysing methods. There were only slight differences in the middle of the ranking list.

Discussion:
Not enough experts participated in this step of expert elicitation to draw significant conclusions. Answers for regions were very much alike; for reservoirs there were more differences between experts.
Conclusion "Conjoint analysis":
The region of origin of a CPE reservoir was considered more important than the reservoir itself, with Asia and Africa as most important regions. The top 3 of most important reservoirs ánd regions was equal in part 1 (choice base conjoint analysis) and part 3 (giving points to reservoirs), which means that experts were consistent in their answers.
People immigrating from abroad to The Netherlands were considered the most important "type" of human. The most important imported animal species were veal calves, followed by dogs and pigs.

3. Workshop to work out pathways in more detail
Date: December 3rd, 2018
Location: Utrecht, Faculty of Veterinary Medicine
Present (all Dutch experts):
- Nedzib Tafro, NVWA, importcontroles dieren en dierlijke producten op Schiphol (zendingen uit derde landen).
- Heike Schmidt, Centrum Zoönosen en Omgevingsmicrobiologie, RIVM en Universiteit Utrecht, AMR in water en mest
- Engeline van Duijkeren, clusterleider binnen het Centrum Zoönosen en Omgevingsmicrobiologie, RIVM, transmissie van resistentie tussen dier en mens, zowel food-borne als direct
- Arjan van Dijk, Nevedi, programmamanager veevoer; heeft voorheen bij Nepluvi gewerkt
- Alex Spieker, Avined, coördinatie van gezondheidszorg in diverse programma's, AI, monitoring van ziekten

Dik Mevius, WBVR en UU, projectteam
Arjan Stegeman, UU, projectteam
Natcha Dankittipong, UU, AIO in BEWARE project (spreekt (nog) geen Nederlands)
Jantien Backer, RIVM, projectteam
Manon Swanenburg, WBVR, projectteam
Clazien de Vos, WBVR, projectteam

<u>Introduction by Arjan Stegeman:</u>
Arjan Stegeman presented the aims and design of the BEWARE project. It consists of four workpackages: 1: Introduction risk of AMR (CPE) into Dutch livestock (pigs, poultry, veal calves)
2: Transmission of AMR within and between farms
3: Developing of an assay for sensitive and specific metagenomics detection of CPE
4: Developing of an early detection surveillance framework using a dynamic mathematical model
Next to BEWARE there is another project (WOT, which means it is paid by the government), carried out at WBVR, in which introduction risks of CPE are determined and suggestions for more efficient surveillance will be done. In this project also companion animals (cats, dogs, horses) are included.

Aim of the workshop:
To get more detailed knowledge about CPE reservoirs, and the pathways/routes from reservoirs to Dutch livestock. Another aim is to rank pathways for their importance.
Presentation workpackage 1:
Manon presents the plan for workpackage 1 of BEWARE: make an inventory of all reservoirs and routes that might contribute to the introduction of CPE in animals in The Netherlands. Reservoirs from abroad but also from within The Netherlands are taken into account. The aim for this work package is to rank the pathways, to identify the most important.
Ga je pdf van presentatie ook meesturen?
Introduction round:
Everybody shortly introduces him/herself. Participants attach a yellow sticky paper to the general model to indicate where their expertise is.
Project results until now:
Manon presents results of earlier expert elicitation rounds about reservoirs and introduction routes. There were 2 earlier expert elicitation rounds. This workshop is the 3rd round of expert elicitation.
Results of the first and second round are described in this report (see page 2-6).
Some remarks were made in response to the results of the first expert elicitation:
- Arjan: (raw) animal products can only be processed in pet food; in livestock feed, fish meal might be used, but that is not raw (example: PAPS, these have undergone a processing step = risk reduction).
- Heike: waste water and hospital water cannot be distinguished from eachother; they are both processed via waste water treatment plant. The original reservoir of CPE are often humans.
- The participating experts say that they don't see water as a reservoir, but as a pathway. Humans are the reservoir, waste water is the route.
- Better definitions of reservoir and pathway (this was not further worked out during the workshop)
Remarks to the results of the second round (conjoint analysis):
- Engeline: was not able to fill in the conjoint analysis. She missed context and definitions.
- Engeline: the answer depends on how risk is defined. Is it for pigs, calves, etc?
- In the conjoint analysis imported animals/products were ranked as less important than in the first expert elicitation round. The workshop participants think this is logical: people are the most important risk for introduction into The Netherlands, and therefore most probably also for introduction into livestock. CPE has only seldomly been detected in livestock so far. However, imported animals might have the highest risk of having (direct) contact with livestock, but how big is this risk?
- Dik: the region North America is perhaps defined too broad. In the USA many CPE have been found. The Netherlands does not import many animals from the USA, but there is substantial import of horses.
There is a discussion about the variability (between experts) in the answers of the conjoint analysis. Ideally we would like to have more participants, but the question is if that is useful, or that the general trend will look the same.

Active participation of experts, drawing pathways:
The participating experts worked in groups (3-4 persons) to try to draw pathways from reservoir to Dutch livestock and pets. This was done in 3 rounds; per round 2 schemes were drawn (2 sectors). Group members changed each round, so that for each sector the "own" expert was in the group.
import of veal calves was originally planned, but was not done, because the veal calf expert could not come. Manon

will ask him to help with that on a later occasion.

Other pathways that will not be worked out during the workshop will be checked by experts who could not come, like the manure pathway (Paul Hoeksema). Arjan also suggests to contact Cumela (?) for manure.

Round 1: import pigs and import poultry

See photo for the results

Extra notes/remarks (not in the schemes):

- There is no testing in pigs for AMR (did they mean at the border or in general??)
- There are no health criteria/demands for AMR at import of animals from EU or 3rd countries.
- Most countries do not have surveillance for CPE. Therefore prevalences in animals are unknown.
- From 3rd countries only import of breeding material (does that mean sperm, ova, or also breeding animals??)
- Is genetic material a risk for AMR/CPE transmission? (antibiotics are added to sperm, gentacide (??)).
- Do pigs come via "collecting locations"? In the Netherlands we don't have them anymore. What about other countries? Check with sector/NVWA.
- Imported pigs are going to the slaughterhouse or a farm in The Netherlands. From the slaughterhouse CPE/AMR can spread to humans via direct contact (slaughterhouse personnel) or consumption of animal products. There is also waste water that can go into the environment.

Side remark (other subject): Nedzib considers import of ornamental fish and fish products and shellfish a high risk. In water of ornamental fish many antibiotics were found (project with Olga Haenen). The water that is imported with the fish is discharged into the drain/sewer in The Netherlands. At Schiphol, CPE have been found in fish products. Dozens of consignments of fish products a day are imported.

Round 2: water and imported feed

Extra notes for water:

- Households and hospitals discharge their water at the same WWTP (waste water treatment plant)
- Water of WWTP is discharged on rivers (surface water); this can also be small rivers; water in sloten (little canals between grass land) is also partly originating from rivers.
- Overflow drain/sewer
- Households with separated waterflow: in about 2% the connection is constructed wrongly, and the waste water comes directly into the surface water.
- Also surface water from abroad via rivers.
- surface water
- Surface water is mainly drunk by animals that stay outside: horses, dairy cattle (partly), sheep. Poultry always gets tap water (strict rules for drinking water), and also pigs and veal calves. Tap water can originate from an own well, but in general this water is clean (filtration by sand).
- Drinking water from the tap almost contains no risk, after treatment for drinking water production.
- Exposure to CPE via surface water also for pets and humans (direct contact, taking in). Indirect exposure via humans to livestock. Pets can also contaminate the surface water.
- CPE are in surface water already; source is humans
- According to an ESBL study: waste water contributes to 60% of the risk (human risk??)
- Travellers

Reizigers veel groter risico voor CPE en ESBL (vormen zij een risico, of lopen zij een risico?). Groter risico dan

- Exposure from surface water is in general low; low concentrations, not much drinking from it, intake is only few CFU per intake. Meat is a much higher risk for humans.

- Dik: "evolutionary risks" ("evolutionaire risico's") --> in case of CPE surface water might play a role, because it is not spread widely. But for ESBL's the contribution of surface water is very low, because other sources became more important.

Round 3: Travellers, import manure and import of pets (as an extra)

Notes for travellers:

- Two groups of people: general population and people who visit farms/work at farms professionally. In this last category we can distinguish between people who only come at one or a low number of farms (farmers, agricultural workers) and people who visit many farms (for example veterinarians).
- The general population has direct contact with pets and animals at "kinderboerderijen" and "zorgboerderijen" .
- The professional workers have direct contact with livestock
- The other route from travellers to livestock is from travellers via surface water to animals.
- risk depends on type of traveller: from which country, hospitalized or not, length of travel,
- It is assumed that the probability of becoming a carrier is higher if you have been a longer period abroad.
- Having "travellers diarrhoea"(with and without treatment) is a risk for being ESBL carrier (I think it was meant to say that it is a risk factor).
- Migrants that regularly travel to and from their home country are considered as a bigger risk.
- Travellers (migrants, many from eastern Europe) working in slaughterhouses might be a risk for contaminating the meat. This is a delicate point (ethnicity of slaughterhouse personnel). This cannot be externally communicated.
- When people are hospitalized, it is not checked if they have been abroad (it is asked if they have been in a hospital abroad or if they have been in contacts with pigs). Having been abroad is a high risk and should be part of the protocol.

Completion

The participants are asked to (again) list a top 3 of reservoirs/pathways that have the highest risk for introduction/exposure of CPE in Dutch animals. This top 3 is separately made for livestock and for pets, and is written on yellow sticky papers, which are attached to the pathways that were drawn during the workshop. The results were not analysed during the workshop.

After the workshop we analysed the results of these rankings.

For livestock the most often mentioned pathways were 1. Water, 2. Import poultry, 3. Travellers (these were also the 3 pathways with the lowest average ranking number).

For pets the most often mentioned pathways were: 1/2 (equal). Import pets/import pet food, 3. Travellers (import pets and pet food had the lowest average ranking number).

Evaluating discussion

Pathways have been sketched. When starting to work them out in a risk model it will probably turn out that more steps per block are needed. Getting real data for filling the model will be a problem in many cases.

Another point for discussion is Wat doe je met de impact van waar de CPE terechtkomt?

Drawing the pathways was a useful exercise, especially because of the presence of different expertises.

The pathway of the veal calf sector has to be made. This sector is considered as a bigger risk for introduction of CPE than the poultry or pig sector.

Conclusion:

Water is a very important factor in the spread/transmission of CPE/AMR after introduction into The Netherlands. In many pathways it is part of the risk. Water in itself is not a reservoir. (The environment was often mentioned as a reservoir; we did not discuss what to do with it).

Supplementary File S3: veal calves' CPE sample size inference.

For countries that had no data on surveillance in calves, prevalence estimates were based on surveillance in bovine meat. In this approach we assumed that ratios of ESBL prevalence between veal calves and in bovine meat of individual MS were similar within the same EU regions and that the ratios of CPE prevalence between veal calves and bovine meat were similar to the ratios of ESBL prevalence between veal calves and bovine meat. Consequently, we inferred the number of veal calves sampled for CPE in MS by comparing **ESBL** prevalence in veal calves to ESBL in bovine meat from available MS:

$$N_{CPE_{sampleVC}} = N_{CPE_{sampleM}} * \frac{P_{ESBL_M}}{P_{ESBL_{vc}}}$$

Where $N_{CPV_{sampleVC}}$ was the expected number of veal calve samples collected to monitor CPE in individual Member States, $N_{CPE_{sampleM}}$ was the number of bovine meat samples collected to monitor CPE in individual Member States, P_{ESBL_M} was the proportion ESBL positive in bovine meat detected in Member States, and $P_{ESBL_{vc}}$ was the proportion ESBL positive in veal calves detected in all available Member States denoted [1].

In both CPE and ESBL surveillance, only 9 EU Member States and 2 EFTA countries have monitored ESBL and CPE in veal calves. Sample size and number of ESBL positive in bovine meat and veal calves from individual Member States (with available veal calve sample) was pooled together based on UN geoscheme regions (West, South, East, and North), P_{ESBL_M} and $P_{ESBL_{vc}}$ (Table S1.1). We used this pooled data based on the same regions to infer sample sizes for veal calves for countries that did not collect any samples from veal calve. For countries in East region, we used the pooled prevalence of ESBL in calves in the other regions because no veal calf samples were collected in any countries of the region (Table S1.2).

Table S1. Proportion ESBL positive in bovine meat and in calves and their ratio for 4 UN regions in EU. Regions highlighted blue are regions with some available veal calves data. East EU data derived from pooled data from all regions.

Regions	P_{ESBL_M}	$P_{ESBL_{vc}}$	$P_{ESBL_M}/P_{ESBL_{vc}}$
West EU	0.023127753	0.47554698	0.049
South EU	0.078698846	0.49366086	0.159
North EU	0.020304569	0.18801997	0.108
East EU	0.038392857	0.4316652	0.089

Table S2. CPE sample size in veal calves inferred from ESBL samples. From left: a) EU region b) EU Member States and EFTA countries c) bovine meat samples collected from each country d) veal calve samples collected from each country, Green: countries with no veal calves sampled and was inferred by multiplying samples collected in bovine meat with $\frac{P_{ESBLM}}{P_{ESBL_{vc}}}$.

		CPE Sample size	
Regions	Countries	bovine meat	<1 yr calf

West	Austria	297	303
West	Belgium	300	300
West	France	302	299
West	Germany	399	349
West	Luxemburg	26	1
West	Netherlands	486	302
West	Switzerland	299	304
East	Bulgaria	150	13
East	Czech republic	301	27
East	Hungary	184	16
East	Poland	300	27
East	Romania	146	13
East	Slovakia	150	13
South	Croatia	369	354
South	Cyprus	139	22
South	Greece	62	10
South	Italy	272	319
South	Malta	300	48
South	Portugal	220	289
South	Slovenia	151	24
South	Spain	300	300
North	Estonia	150	38
North	Finland	324	315
North	Ireland	300	32
North	Latvia	149	16
North	Lithuania	150	16
North	Sweden	286	31
North	United Kingdom	314	34
North	Iceland	95	10
North	Norway	343	303
North	Denmark	292	297

Supplementary File S4: Community - Clinical prevalence

Probability of acquiring CPE from the community versus clinical during holiday was expected to be different since sources of CPE (patients) in the hospital setting is more saturated than CPE sources in the community (healthy adults, food contamination), and exposure time to CPE sources would be different. Given that we only have prevalence data from the clinical setting, we inferred community prevalence by a) using correlation between community and clinical setting in ESBL, b) using colonization period and travel times to narrow down exposure period of community CPE.

The probability of acquiring CPE from the clinical setting, $\boldsymbol{P_{CPE_m}}$, is $BETA(Positive_{pooled} + 1, Sample_{pooled} - Positive_{pooled} + 1)$ and the probability of acquiring CPE from the community was inferred from $\boldsymbol{P_{CPE_m}}$ multiplied by the correlation coefficient of ESBL in the community versus ESBL in the clinical setting, $\boldsymbol{CoefESBL_{com:cli}}$, (0.79) because we assumed the correlation between CPE from the community and clinical prevalence would follow the trend of ESBL.

The correlation coefficient of ESBL in the community versus ESBL in the clinical setting was calculated using 5 publications from EU Member States (Table S2.1). We applied clinical and community prevalence from the same year in the Pearson correlation test to calculate the correlation between community ESBL and clinical ESBL.

Table S3. ESBL prevalence in community and clinical setting collected from the literature review.

Regions	Countries	Years	Clinical prevalence	Community prevalence	References
EU_east	Czech republic	2010	2.1	0.4	[2]
EU_north	Ireland	2006	0.0	0.0	[3]
EU_north	Ireland	2007	0.0	0.0	[3]
EU_north	Ireland	2008	0.0	0.0	[3]
EU_north	Ireland	2009	0.0	0.0	[3]
EU_north	Ireland	2010	0.0	0.0	[3]
EU_north	Ireland	2011	0.0	0.0	[3]
EU_north	Ireland	2012	0.1	0.0	[3]
EU_north	Ireland	2013	0.1	0.0	[3]
EU_north	Ireland	2014	0.1	0.0	[3]
EU_west	Belgium	2006	0.2	0.1	[4]
EU_west	Belgium	2008	0.2	0.0	[5]
EU_west	Denmark	2009	0.1	0.0	[6]

Apart from the lesser probability of acquiring community CPE, we also calculated the exposure duration to community CPE. While exposure duration to nosocomial CPE would be total time spent in the hospital, the exposure duration to community CPE would be the total time spent on holiday per year. We calculated CPE incidence rate per day, which was the prevalence of CPE in hospital, $Prev_{CPE_m}$, divided by average duration of colonization, $T_{days_{CPE}}$. We then multiplied this incidence rate by the average number of days of holiday abroad taken by Dutch citizens, T_{travel}.

$$P_{cCPE_m} = \frac{\boldsymbol{P_{CPE_m}} * CoefESBL_{com:cli}}{T_{days_{CPE}}} * T_{travel}$$

Supplementary File S5: Estimated CPE in local and imported companion animals

We estimated the number of local companion animals (dog, cat) in the Netherlands, including housed animals and stray animals using data reported by the National Institute for Public Health and the Environment (RIVM) and Wageningen University & Research reports [7, 8]. We further estimated the imported companion animals from other EU regions, including stray animals and animals from commercial breeders, using reports from the Stray Animal Foundation and BUZhonden website[8, 9]. To estimate the number of CPE-colonized companion animals in the farms with animal *i*, we first calculated the number of CPE-colonized companion animals in the Dutch companion animal population. Total number of local companion animals was multiplied by human CPE prevalence in the Netherlands, while the imported number of companion animals from EU regions was multiplied by human CPE prevalence of the same EU regions. Furthermore, we calculated the prevalence of CPE in companion animals of different sources (local & imports) by dividing the number of CPE-colonized companion animals by total companion animals. Subsequently, we estimated the number of farms with CPE-colonized companion animals (of different origin) by multiplying the individual CPE prevalence of different origin by the number of farms with companion animals.

Table S4. Components for calculation of N_{colC_i} (preliminary). From left, a) Companion animals' countries of origin, b) CPE prevalence in humans, c) estimated number of colonized companion animals in Dutch companion animal population, d) estimated number of colonized companion animal in farms.

**Origin**	**No. dogs in NL**	**CPE_prev_human**	**No. colonized dogs**	**No. farms with colonized dog**
NL	1,500,000	0.0011 (0.0004, 0.002)	1,628 (587, 2359)	12 (4, 18)
East	83,348	0.0004 (0.0004, 0.001)	35 (34, 114)	0 (0, 1)
West	20,202	0.0008 (0.0006, 0.001)	17 (14, 22)	0
South	7,616	0.0030 (0.002, 0.0034	23 (18, 27)	0
Origin	No. cats in NL	CPE_prev_human	No. colonized cats	No. farms with colonized cat
NL	2,299,566	0.0011 (0.0004, 0.002)	2,495 (900, 3617)	13 (7, 28)
East	120,221	0.0004 (0.0004, 0.001)	50 (49, 164)	0 (0, 1)
West	30,000	0.0008 (0.0006, 0.001)	25 (20, 32)	0
South	664	0.0030 (0.002, 0.0034)	2 (2, 2)	0

Supplementary File S6: Model input

Table S5. Inputs to estimate the number of farms exposed to CPE.

**Parameters**	**Input parameter**	**Value (default)**	**Unit**	**Data source**
N_{farm_i}	Number of broiler farms in the Netherlands	625	Farms	[10]
	Number of broiler breeder farms in the Netherlands	272	Farms	
	Number of pig-fattening farms in the Netherlands	2910	Farms	
	Number of pig-breeding (with piglets) farms in the Netherlands	1196	Farms	
	Number of farrow to finish pig farms in the Netherlands	640	Farms	
	Number of veal calves fattening farms in the Netherlands	1667	Farm	
N_{animal_i}	Number of broilers in the Netherlands	45230035	Animals	[10]
	Number of broiler parents in the Netherlands	8815525	Animals	
	Number of fattening pigs in the Netherlands	5211511	Animals	
	Number of breeding pigs in the Netherlands	1129564	Animals	
	Number of veal calves in the Netherlands	898107	Animals	
N_{vet_i}	Number of veterinarians working with broilers	109	Veterinarians	[10]
	Number of veterinarians working with parent broilers	109	Veterinarians	
	Number of veterinarians working with fattening pigs,	275	Veterinarians	
	Number of veterinarians working with breeding pigs and piglets	275	Veterinarians	
	Number of veterinarians working with farrow to finish farm	275	Veterinarians	
	Number of veterinarians working with veal calves	155	Veterinarians	
P_{T_m}	Proportion of Dutch citizens traveling to South Asia	0.003	Fraction	[10, 11]
	Proportion of Dutch citizens traveling to Central and East Asia	0.005	Fraction	
	Proportion of Dutch citizens traveling to Western Asia	0.06	Fraction	
	Proportion of Dutch citizens traveling to Northern Africa	0.023	Fraction	

	Proportion of Dutch citizens traveling to Southeast Asia	0.014	Fraction	
	Proportion of Dutch citizens traveling to Central America and Caribbean	0.012	Fraction	
	Proportion of Dutch citizens traveling to Central and Eastern Africa	0.003	Fraction	
	Proportion of Dutch citizens traveling to western Africa	0.004	Fraction	
	Proportion of Dutch citizens traveling to Southern America	0.004	Fraction	
	Proportion of Dutch citizens traveling to Southern Africa	0.003	Fraction	
	Proportion of Dutch citizens traveling to Western Europe	0.43	Fraction	
	Proportion of Dutch citizens traveling to Southern Europe	0.243	Fraction	
	Proportion of Dutch citizens traveling to Northern Europe	0.064	Fraction	
	Proportion of Dutch citizens traveling to Eastern Europe	0.022	Fraction	
P_{CPE_m}	Probability of acquiring CPE in a hospital in Southern Asia	BETA (4,587, 22,205)	Fraction	19-12月
	Probability of acquiring CPE in a hospital in Central and Eastern Asia	BETA (11,879, 215,059)	Fraction	12, 20, 21
	Probability of acquiring CPE in a hospital in Western Asia	BETA (1,868, 19,110)	Fraction	12, 22-24
	Probability of acquiring CPE in a hospital in Northern Africa	BETA (34, 610)	Fraction	12
	Probability of acquiring CPE in a hospital in Southeast Asia	BETA (18,066, 261,116)	Fraction	12, 25-28
	Probability of acquiring CPE in a hospital in Central America and Caribbean	BETA (538, 17,162)	Fraction	12
	Probability of acquiring CPE in a hospital in Central and Eastern Africa	BETA (3, 1,056)	Fraction	12
	Probability of acquiring CPE in a hospital in Western Africa	BETA (10, 107)	Fraction	12
	Probability of acquiring CPE in a hospital in Southern America	BETA (729, 13,172)	Fraction	12, 29
	Probability of acquiring CPE in a hospital in Southern Africa	BETA (851, 1,554)	Fraction	12, 30
	Probability of acquiring CPE in a hospital in Western Europe	BETA (113, 66,129)	Fraction	31
	Probability of acquiring CPE in a hospital in Southern Europe	BETA (2,066, 28,171)	Fraction	31
	Probability of acquiring CPE in a hospital in Northern Europe	BETA (53, 58,021)	Fraction	31
	Probability of acquiring CPE in a hospital in Eastern Europe	BETA (437, 13,888)	Fraction	31
	Probability of acquiring CPE in a hospital in Northern America	BETA (998, 9,979)	Fraction	12, 32, 33
	Probability of acquiring CPE in a hospital in Oceania	BETA (192, 2,925)	Fraction	12
c_{a_i}	Grams consumed per broiler per day	79	Average grams	34
	Grams consumed per broiler parent per day	39	Average grams	34
	Grams consumed per fattening pig per day	3,500	Average grams	35
	Grams consumed per sows per day	4,000	Average grams	35
	Grams consumed per rose veal calf per day	3,917	Average grams	36

Grams consumed per blank veal calf per day	1,429	Average grams	37

Supplementary File S7 : Queries to retrieve import livestock of interest from cbs.nl

URL: https://opendata.cbs.nl/statline/portal.html

- Click "Kies thema" at the top of the page
- Click the following options internationale handel> handel; goederen> goederensoorten, landen per jaar> natuur, voeding en tabak; jaar
- Click "Preview data" then Select the following animal species from the drop down "goederensoorten natuur, voeding en tabak"

  0102291000: Cattle, live, with a weight of <= 80 kg (excl. pure-bred breeding cattle)

  0102900500: Cattle/ domestic animals/live weighing <= 80 kg (excl. pure-bred breeding animals)

  0103100000: Pure-bred breeding pigs

  0103911000: Pigs/ domestic animals/ live pigs weighing <50 kg (excl. pure-bred breeding animals

  0103921100: Sows /domestic animals /live ..."who have farrowed at least once, weighing> = 160 kg (excl. pure-bred breeding animals)

  0105111900: Female breeding chicks of chickens/ poultry/weighing <= 185 g (excl. those of laying breeds)

  0105119900: Roosters and chickens/ poultry/ weighing <= 185 g (excl. those of laying breeds and excl. female and breeding chicks)
- Specify imported animals by select drop down "Onderwerp" > Invoerhoeveelheid

Specify countries of import (European Member states in our analysis) by select drop down "Landen"

Supplementary File S8: One-at-a-time sensitivity analysis on introduction

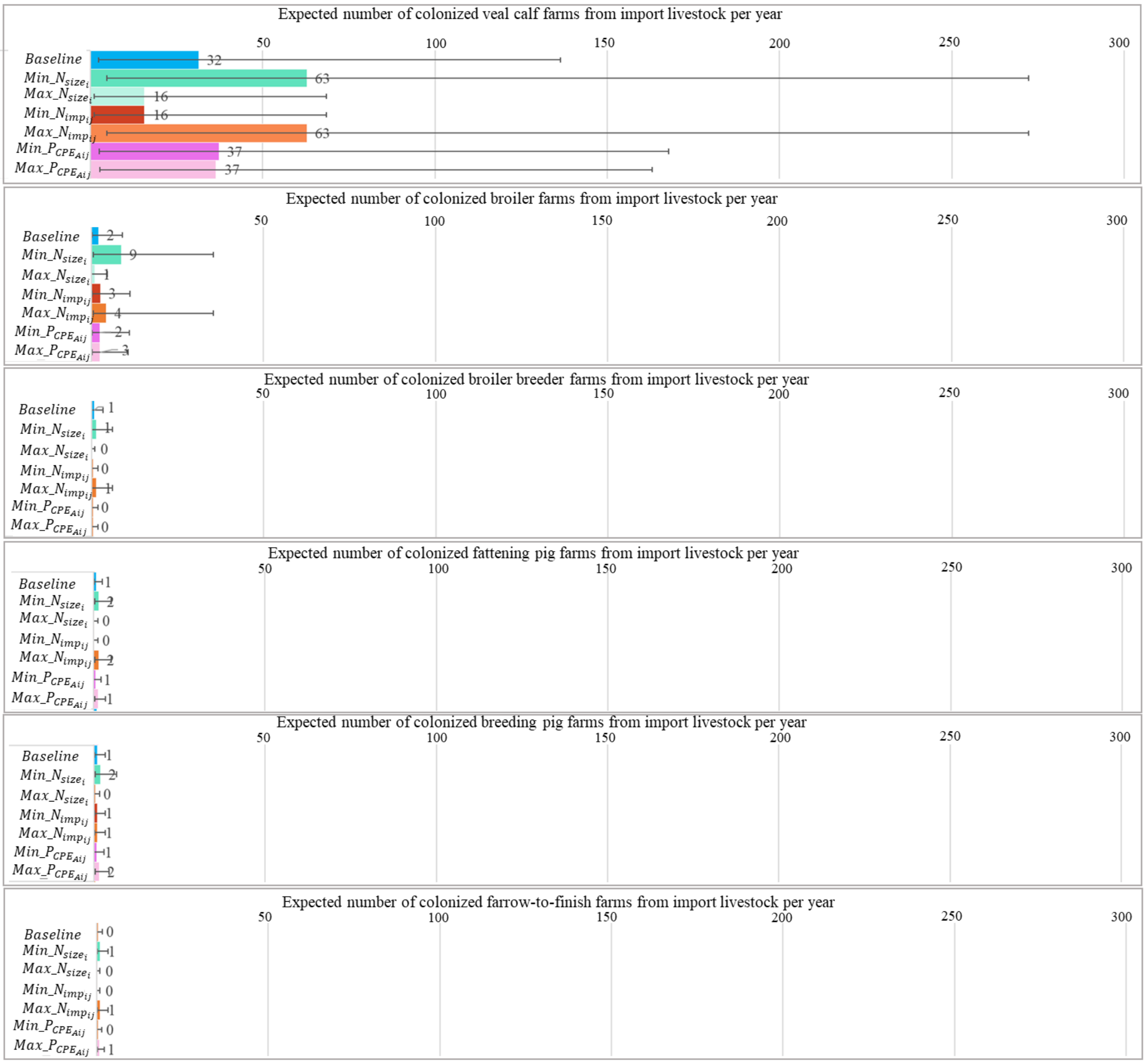

Figure S2. One-at-a-time sensitivity analysis from livestock import source. One input was discounted or raised two-fold in each round. The resulting number of introductions is shown here.

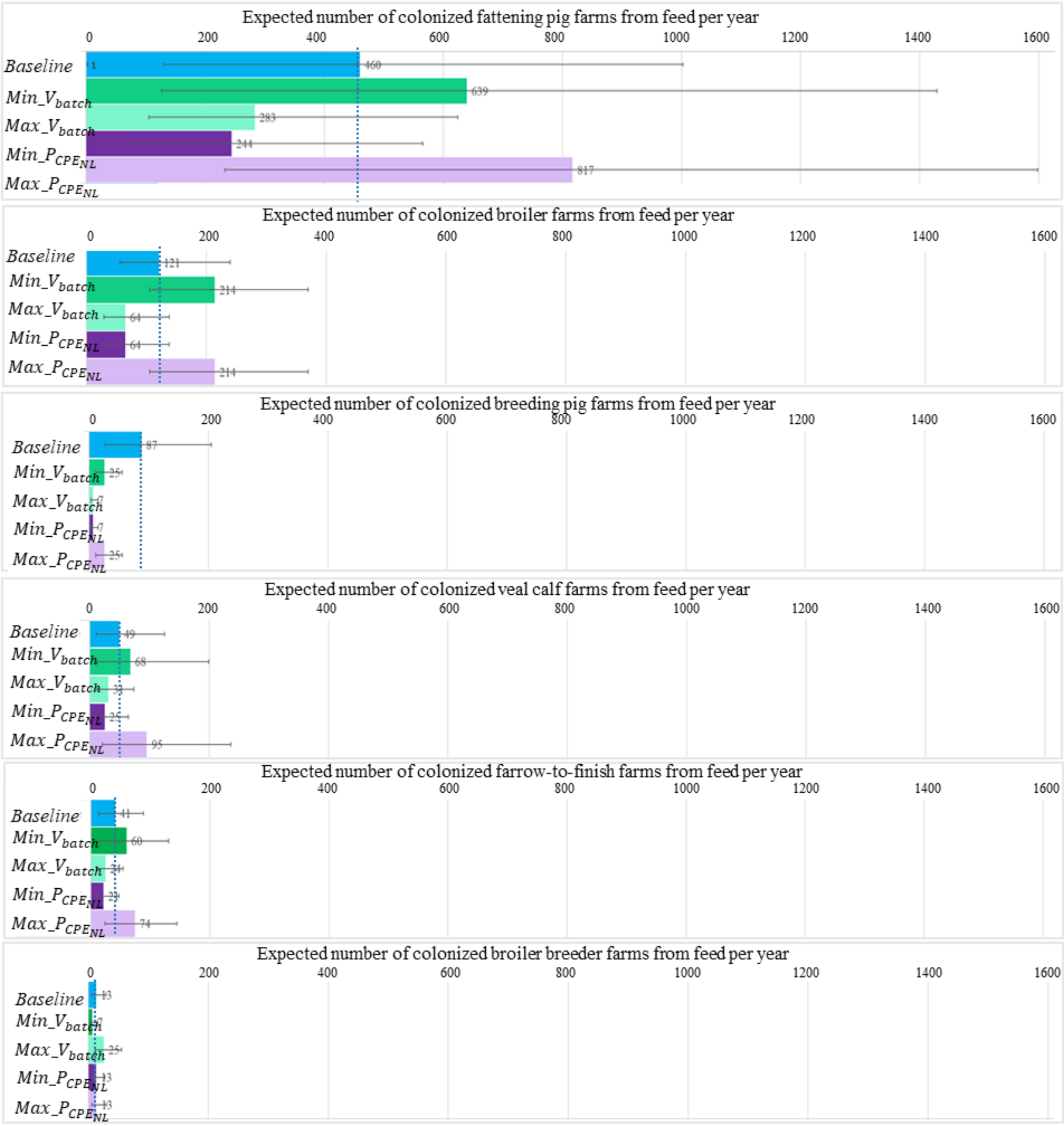

Figure S3. One-at-a-time additional parameters sensitivity analysis of feed source. One input was discounted or raised two-fold in each round. The resulting number of introductions is shown here. These parameters were excluded from the main text (Figure 3) because these parameters have same input in all farm types.

Supplementary File S9: Result of one-at-a-time between sources sensitivity analysis on introduction feed, imported livestock, companion animal, returning traveler, and hospitalized patients. In every source, one input parameter was adjusted in each test and the resulting number of introductions were compared between sources.

Table S6. Total number of test runs in which one parameter was discounted or increased two-fold.

Sources	Total number of test runs	Number of tests in each farm type	Number of introductions in baseline model	Number of introductions in the least risk model	Number of introductions in the highest risk model
Feed	90	15	777	408	1,407
Import	42	7	44	22	87

Companion	102	17	0	0	3
Traveler	156	26	0	0	0
Hospital	156	26	0	0	0
Total	546	91	821	430	1,497

Table S7. Comparison of introduction between livestock feed and imported livestock. Livestock feed remain a higher risk source than import livestock except few tests in veal calf farms.

	Total number of sensitivity tests	Number of tests where ranking remains the same	Probability of rank remains unchanged
Broiler	104	104	1
broiler breeder	104	104	1
fattening pig	104	104	1
breeding pig	104	104	1
farrow-to-finish	104	104	1
veal calf	104	58	0.56
Total	624	578	0.93

Table S8. Comparison of introduction between imported livestock and returning traveler sources. Imported livestock remain a higher risk source than returning traveler. Though, few test resulted in zero introduction from imported livestock to broiler breeder and farrow-to-finish farms which lower the high rank of imported livestock to low.

**Import/traveler***						
	Total number of tests	Number of tests where ranking remains the same	Number of tests where ranking changes	Probability of rank remaining unchanged	Probability of rank changes	Probability of outcome from both sources equal/close to zero
broiler	182	182	0	1	0	0
broiler breeder	182	52	130	0.29	0.71	0.71
fattening pig	182	182	0	1	0	0
breeding pig	182	182	0	1	0	0
farrow-to-finish	182	104	78	0.57	0.43	0.43
veal calf	182	182	0	1	0	0
**Total**	1092	884	208	0.81	0.19	0.19

* There are many parameters overlapped between returning traveler and hospitalized farm worker sources. The result of OAT sensitivity analysis in hospitalized farm workers is mostly identical to returning travelers. Thus, Import/hospitalize result is equivalent to Import/traveler.

Table S9. Comparison of introduction between imported livestock and companion animal sources. Imported livestock have significant probability to produce small introduction in broiler breeder and farrow-to-finish farms that is equal to introduction from companion animals. Proportion of time companion animal spends in barn (P_{barnC}) is the input parameter that started introduction from companion animal.

**import/ companion**						
	Total number of tests	Number of tests where ranking remains the same	Number of tests where ranking changes	Probability of rank remaining unchanged	Probability of rank changes	Probability of outcome from both sources are equal (mostly close to zero)
broiler	119	119	0	1	0	0

broiler breeder	119	34	85	0.28	0	0.72
fattening pig	119	115	4	0.96	0	0.04
breeding pig	119	115	4	0.96	0	0.04
farrow to finish	119	68	51	0.57	0	0.43
veal calf	119	119	0	1	0	0
**Total**	714	570	144	0.79	0	0.20

Supplementary File S10: Introduction from imported livestock to veal calf farms

Table S10. Top six countries with the highest number of introductions from imported livestock to veal calf farm. $N_{intros,i}$ is the median expected number of farms with introduction with upper and lower 95th percentile in brackets, $N_{A_{ij}}$ is number of imported batches of animals, $P_{CPE_{A_{ij}}}$ where expected CPE prevalence is estimated as the upper limit * reported from sampled animals or otherwise inferred from ESBL prevalence. Highlighted boxes are input with the top-five highest values.

Member States	$N_{intros,i}$	$N_{A_{ij}}$	$P_{CPE_{A_{ij}}}$
**Germany**	10 (1, 43)	3293	0.001
**Latvia**	9 (1, 39)	151	0.116
**Ireland**	6 (0, 27)	211	0.033
**Czech republic**	4 (0, 28)	114	0.040
**Lithuania**	3 (0, 13)	54	0.037
**Estonia**	2 (0, 11)	93	0.065
**Belgium**	1 (0, 6)	370	0.002

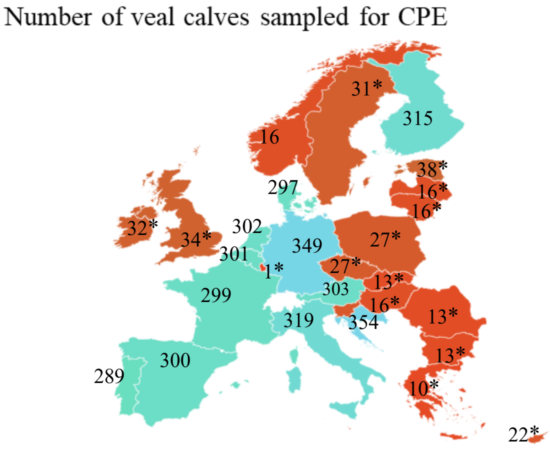

Figure S4. Number of veal calves sampled in the import countries of origin reported by EARS-Net 2018. Number with * is the number of animals inferred from ESBL data in veal calf and bovine meat (Supplementary III). All countries reported zero positive veal calf.

References

1. European Food Safety, A.; European Centre for Disease, P.; Control, The European Union summary report on antimicrobial resistance in zoonotic and indicator bacteria from humans, animals and food in 2016. *EFSA J* **2018,** *16* (2), e05182.
2. Husickova, V.; Cekanova, L.; Chroma, M.; Htoutou-Sedlakova, M.; Hricova, K.; Kolar, M., Carriage of ESBL- and AmpC-positive Enterobacteriaceae in the gastrointestinal tract of community subjects and hospitalized patients in the Czech Republic. *Biomed Pap Med Fac Univ Palacky Olomouc Czech Repub* **2012,** *156* (4), 348-53.
3. Stapleton J, P.; O'Kelly, f.; Lundon J, D.; Lynch, M.; McWade, R.; Scanlon, N.; Hannan, M., Antibiotic resistance patterns of Escherichia coli urinary isolates and comparison with antibiotic consumption data over 10 years, 2005–2014. *Ir J Med Sci* **2017,** *186*, 733-741.

4. Smet, A.; Martel, A.; Persoons, D.; Dewulf, J.; Heyndrickx, M.; Claeys, G.; Lontie, M. V. M., B. ; Herman, L.; Haesebrouck, F.; Butaye, P., Characterization of Extended-Spectrum b-Lactamases Produced by Escherichia coli Isolated from Hospitalized and Nonhospitalized Patients:
Emergence of CTX-M-15-Producing Strains Causing Urinary Tract Infections. *MICROBIAL DRUG RESISTANCE* **2010,** *16* (2).
5. Schoevaerdts, D.; Bogaerts, P.; Grimmelprez, A.; de Saint-Hubert, M.; Delaere, B.; Jamart, J.; Swine, C.; Glupczynski, Y., Clinical profiles of patients colonized or infected with extended-spectrum beta-lactamase producing Enterobacteriaceae isolates: a 20 month retrospective study at a Belgian University Hospital. *BMC Infect Dis* **2011,** *11*, 12.
6. Olesen, B.; Hansen, S. D.; Nilsson, F.; Frimodt-Møller, J.; Leihof, F.; Struve, C.; Scheutz, F.; Johnston, B.; A Krogfelt, K.; Johnsond, R. J., Prevalence and Characteristics of the Epidemic Multiresistant Escherichia coli ST131 Clonal Group among Extended-Spectrum BetaLactamase-Producing E. coli Isolates in Copenhagen, Denmark. *JCM* **2013,** (JournalASMorg).
7. van Heijst, B.; de Kort, I.; Overgaauw, P.; Vinke, C. M.; Beekmans, M. *Feiten & Cijfers Gezelschapsdierensector* Utrecht university: 2015.
8. Radstake, C. *Immigratie van buitenlandse (zwerf)honden & - (zwerf)katten naar Nederland: de cijfers 2016*; Stray Animal Foundation Platform: 2016.
9. Platform, T. A. F. Cijfers. https://buzhonden.weebly.com/cijfers-import.html#.
10. Statistiek, C. B. v. d. Statline. https://opendata.cbs.nl/statline#/CBS/nl/.
11. Arcilla, M. S.; van Hattem, J. M.; Haverkate, M. R.; Bootsma, M. C. J.; van Genderen, P. J. J.; Goorhuis, A.; Grobusch, M. P.; Lashof, A. M. O.; Molhoek, N.; Schultsz, C.; Stobberingh, E. E.; Verbrugh, H. A.; de Jong, M. D.; Melles, D. C.; Penders, J., Import and spread of extended-spectrum β-lactamase-producing Enterobacteriaceae by international travellers (COMBAT study): a prospective, multicentre cohort study. *The Lancet Infectious Diseases* **2017,** *17* (1), 78-85.
12. WHO, ANTIMICROBIAL RESISTANCE Global Report on Surveillance. **2014**.
13. Khan, E., Ejaz, M., Zafar, A., Jabeen, K., Shakoor, S., Inayat, R., Hasan, R., Increased isolation of ESBL producing Klebsiella pneumoniae with emergence of carbapenem resistant isolates in Pakistan: report from a tertiary care hospital. **2010**.
14. Castanheira, M.; Deshpande, L. M.; Mathai, D.; Bell, J. M.; Jones, R. N.; Mendes, R. E., Early dissemination of NDM-1- and OXA-181-producing Enterobacteriaceae in Indian hospitals: report from the SENTRY Antimicrobial Surveillance Program, 2006-2007. *Antimicrob Agents Chemother* **2011,** *55* (3), 1274-8.
15. Mohanty, S.; Gaind, R.; Ranjan, R.; Deb, M., Prevalence and phenotypic characterisation of carbapenem resistance in Enterobacteriaceae bloodstream isolates in a tertiary care hospital in India. *Int J Antimicrob Agents* **2011,** *37* (3), 273-5.
16. Khajuria, A.; Praharaj, A. K.; Kumar, M.; Grover, N., Emergence of Escherichia coli, Co-Producing NDM-1 and OXA-48 Carbapenemases, in Urinary Isolates, at a Tertiary Care Centre at Central India. *J Clin Diagn Res* **2014,** *8* (6), DC01-4.
17. Murali Alagesan1, Ram Gopalakrishnan2, Senthur Nambi Panchatcharam3, Sureshkumar Dorairajan4 Mandayam Ananth5, Ramasubramanian Venkatasubramanian, A decade of change in susceptibility patterns of Gram-negative blood culture isolates: a single center study. **2015**.
18. Hsu, L. Y.; Apisarnthanarak, A.; Khan, E.; Suwantarat, N.; Ghafur, A.; Tambyah, P. A., Carbapenem-Resistant Acinetobacter baumannii and Enterobacteriaceae in South and Southeast Asia. *Clin Microbiol Rev* **2017,** *30* (1), 1-22.
19. Nahid, F.; Khan, A. A.; Rehman, S.; Zahra, R., Prevalence of metallo-beta-lactamase NDM-1-producing multi-drug resistant bacteria at two Pakistani hospitals and implications for public health. *J Infect Public Health* **2013,** *6* (6), 487-93.
20. Liu, S. W.; Chang, H. J.; Chia, J. H.; Kuo, A. J.; Wu, T. L.; Lee, M. H., Outcomes and characteristics of ertapenem-nonsusceptible Klebsiella pneumoniae bacteremia at a university hospital in Northern Taiwan: a matched case-control study. *J Microbiol Immunol Infect* **2012,** *45* (2), 113-9.
21. Liu, J.; Yu, J.; Chen, F.; Yu, J.; Simner, P.; Tamma, P.; Liu, Y.; Shen, L., Emergence and establishment of KPC-2-producing ST11 Klebsiella pneumoniae in a general hospital in Shanghai, China. *Eur J Clin Microbiol Infect Dis* **2018,** *37* (2), 293-299.
22. Ben-David, D.; Kordevani, R.; Keller, N.; Tal, I.; Marzel, A.; Gal-Mor, O.; Maor, Y.; Rahav, G., Outcome of carbapenem resistant Klebsiella pneumoniae bloodstream infections. *Clin Microbiol Infect* **2012,** *18* (1), 54-60.
23. Schwaber, M. J.; Klarfeld-Lidji, S.; Navon-Venezia, S.; Schwartz, D.; Leavitt, A.; Carmeli, Y., Predictors of carbapenem-resistant Klebsiella pneumoniae acquisition among hospitalized adults and effect of acquisition on mortality. *Antimicrob Agents Chemother* **2008,** *52* (3), 1028-33.
24. Al Johani, S. M.; Akhter, J.; Balkhy, H.; El-Saed, A.; Younan, M.; Memish, Z., Prevalence of antimicrobial resistance among gram-negative isolates in an adult intensive care unit at a tertiary care center in Saudi Arabia. *Ann Saudi Med* **2010,** *30* (5), 364-9.
25. Rimrang, B.; Chanawong, A.; Lulitanond, A.; Wilailuckana, C.; Charoensri, N.; Sribenjalux, P.; Phumsrikaew, W.; Wonglakorn, L.; Kerdsin, A.; Chetchotisakd, P., Emergence of NDM-1- and IMP-14a-producing Enterobacteriaceae in Thailand. *J Antimicrob Chemother* **2012,** *67* (11), 2626-30.
26. Balm, M. N.; La, M. V.; Krishnan, P.; Jureen, R.; Lin, R. T.; Teo, J. W., Emergence of Klebsiella pneumoniae co-producing NDM-type and OXA-181 carbapenemases. *Clin Microbiol Infect* **2013,** *19* (9), E421-3.
27. Tran, H. H.; Ehsani, S.; Shibayama, K.; Matsui, M.; Suzuki, S.; Nguyen, M. B.; Tran, D. N.; Tran, V. P.; Tran, D. L.; Nguyen, H. T.; Dang, D. A.; Trinh, H. S.; Nguyen, T. H.; Wertheim, H. F., Common isolation of New Delhi metallo-beta-lactamase 1-producing Enterobacteriaceae in a large surgical hospital in Vietnam. *Eur J Clin Microbiol Infect Dis* **2015,** *34* (6), 1247-54.

28. thailand, N., ercentage of susceptible Organisms Isolated From All Specimen, 85 hospitals, CPE_thailand. **2018**.
29. Luci Correa1, Marines Dalla Valle Martino2, Itacy Siqueira2, Jacyr Pasternak2, Ana Cristina Gales3, Claudia Vallone Silva1, Thiago Zinsly Sampaio Camargo4, Patricia Faria Scherer4 and Alexandre Rodrigues Marra4, A hospital-based matched case–control study to identify clinical outcome and risk factors associated with carbapenem-resistant Klebsiella pneumoniae infection. **2013**.
30. Singh-Moodley, A.; Perovic, O., Antimicrobial susceptibility testing in predicting the presence of carbapenemase genes in Enterobacteriaceae in South Africa. *BMC Infect Dis* **2016,** *16* (1), 536.
31. ECDC, Annual report of the European Antimicrobial Resistance Surveillance Network (EARS-Net). *SURVEILLANCE REPORT* **2017**.
32. Iregui, A.; Ha, K.; Meleney, K.; Landman, D.; Quale, J., Carbapenemases in New York City: the continued decline of KPC-producing Klebsiella pneumoniae, but a new threat emerges. *J Antimicrob Chemother* **2018,** *73* (11), 2997-3000.
33. Patel, G.; Huprikar, S.; Factor, S. H.; Jenkins, S. G.; Calfee, D. P., Outcomes of carbapenem-resistant Klebsiella pneumoniae infection and the impact of antimicrobial and adjunctive therapies. *Infect Control Hosp Epidemiol* **2008,** *29* (12), 1099-106.
34. Animals, T. E. C. f. F. *THE WELFARE OF BROILER CHICKENS IN THE EUROPEAN UNION*; 2005.
35. Rönnqvist, M.; Välttilä, V.; Heinola, K.; Ranta, J.; Niemi, J.; Tuominen, P. *Risk assessment and cost–benefit analysis of salmonella in feed and animal production*; Ministry of Agriculture and Forestry: 2018.
36. Bussel, V. Veal farm. https://vanbusselbv.nl/en/veal-farm/.
37. van Doremalen, N.; Bushmaker, T.; Karesh, W. B.; Munster, V. J., Stability of Middle East respiratory syndrome coronavirus in milk. *Emerg Infect Dis* **2014,** *20* (7), 1263-4.
